# Magnetic resonance imaging findings in patients with idiopathic olfactory dysfunction and normal findings on nasoendoscopy

**DOI:** 10.1017/S0022215122000913

**Published:** 2023-01

**Authors:** I M Tung, R Misirovs, Q Gardiner

**Affiliations:** 1 Ninewells Hospital and Medical School, NHS Tayside, Dundee, Scotland, UK; 2 Department of ENT, Ninewells Hospital and Medical School, NHS Tayside, Dundee, Scotland, UK

**Keywords:** Anosmia, Olfaction Disorders, Esthesioneuroblastoma, Magnetic Resonance Imaging, Smell

## Abstract

**Objective:**

In presentations of anosmia or dysosmia, magnetic resonance imaging may be required to screen for intracranial pathology such as olfactory neuroblastomas and other intracranial masses impacting on the olfactory pathway. This study aimed to establish positive magnetic resonance imaging findings of anosmia or dysosmia for scans performed before the coronavirus disease 2019 pandemic.

**Methods:**

The study examined the outcome of patients who presented with isolated olfactory dysfunction and who underwent magnetic resonance imaging between 2015 and 2019.

**Results:**

Of the 131 patients, 41 (31.3 per cent) had normal scan findings, 50 (38.2 per cent) had insignificant paranasal mucosal disease and 6 (4.6 per cent) had mucosal thickening significant enough to require additional intervention. These interventions included repeat nasoendoscopy or commencement of intranasal or oral steroids. No patients had olfactory neuroblastoma.

**Conclusion:**

Only 4.6 per cent of the magnetic resonance imaging scans revealed abnormal findings related to anosmia or dysosmia, and none required ENT surgical intervention. None of the magnetic resonance imaging scans identified an olfactory neuroblastoma or intracranial masses impacting on the olfactory pathway.

## Introduction

Olfaction has many important roles in our daily lives, including roles in: maintaining good nutritional health, sensation of pleasure, interpersonal behaviour, and identifying dangerous compounds such as expired food, smoke, dangerous chemicals and so on.^1^ Hence, when a person experiences olfactory dysfunction, not only does it impact their quality of life, but it could also potentially be a danger to their health and safety.

Numerous studies have measured the prevalence of olfactory disorders. A meta-analysis by Yang and Pinto showed a variable prevalence depending on the study population and demographics.^1^ Prevalence also varies depending on whether the dysfunction is self-reported or objectively measured. Self-reported prevalence varies from 1.4 per cent to 15.3 per cent; when based on objective assessment, the prevalence of olfactory dysfunction varies from 2.7 per cent to 24.5 per cent.^1^

Olfactory dysfunction can be broadly classified as qualitative or quantitative. Quantitative olfactory dysfunction refers to a diminished function of smell (hyposmia) or a complete loss of smell (anosmia). Qualitative olfactory dysfunction refers to an altered sense of smell. There are two common representations of qualitative olfactory dysfunction. The first is parosmia, where people often perceive smells to be unpleasant, such as rotting, burning or foul-smelling odours.^2,3^ The second is phantosmia, where people perceive the presence of an odour in the absence of that odour.

There are many causes of olfactory dysfunction, the most common being viral respiratory infection, rhinitis, medications, nasal polyps, deviated nasal septum and intracranial trauma. Olfactory dysfunction is now also recognised as a symptom of coronavirus disease 2019 (Covid-19).

In the absence of any obvious precipitating factors, and with normal nasal endoscopy examination findings, a magnetic resonance imaging (MRI) scan is performed to rule out intracranial lesions such as olfactory neuroblastoma and meningiomas that may be affecting the olfactory pathway. Although the incidence of olfactory neuroblastoma is only 0.4 per million of the population,^4^ it is a diagnosis that should be excluded because of its potential to cause harm.

Our study looked at the MRI scans performed for these patients before the Covid-19 pandemic, and determined whether the scans resulted in any interventions and identified what those interventions were.

## Materials and methods

This is a retrospective study based at Ninewells Hospital, Dundee, Scotland. The scans were completed between January 2015 and December 2019. The reports were obtained from the radiology department. We first looked at all scans requested by ENT consultants. Search terms, including anosmia, hyposmia, parosmia, phantosmia, smell and olfactory, were used to identify those MRI scans requested for patients presenting with olfactory dysfunction.

The MRI scans were performed to rule out an intracranial cause, such as olfactory neuroblastoma or meningiomas, which might be impacting on the olfactory pathway, in patients with no abnormal findings on nasoendoscopy. The reports given by the radiology department were reviewed. The MRI findings were then grouped and categorised.

The follow-up plans for the patients were obtained by reviewing the clinic letters after the scanning was performed. Based on the discussion and outcome of the follow-up clinics, the radiological findings were categorised as either incidental or relevant. The MRI results that showed that mucosal thickening was classified as insignificant if there was no follow up, and was considered relevant if any intervention or follow up was required.

## Results

A total of 131 patients underwent an MRI scan for their olfactory dysfunction within the five-year study period. Seventy-one patients (54.2 per cent) were female (Table 1). Seventy-nine patients (60.3 per cent) complained of anosmia, 30 (22.9 per cent) presented with parosmia, 20 (15.3 per cent) complained of hyposmia and 8 (6.1 per cent) presented with phantosmia (Table 1).

**Table 1.** Patient demographics*

Parameter	Values
Age (mean ± SD; years)	55.3 ± 15.9
Sex (*n* (%))	
– Male	60 (45.8)
– Female	71 (54.2)
Presenting complaint (*n* (%))^†^	
– Anosmia	79 (60.3)
– Hyposmia	20 (15.3)
– Parosmia	30 (22.9)
– Phantosmia	8 (6.1)

* Total *n* = 131. ^†^Cumulative frequency exceeds 100 per cent as some scan requests mentioned more than one presenting complaint. SD = standard deviation

**Fig. 1.**
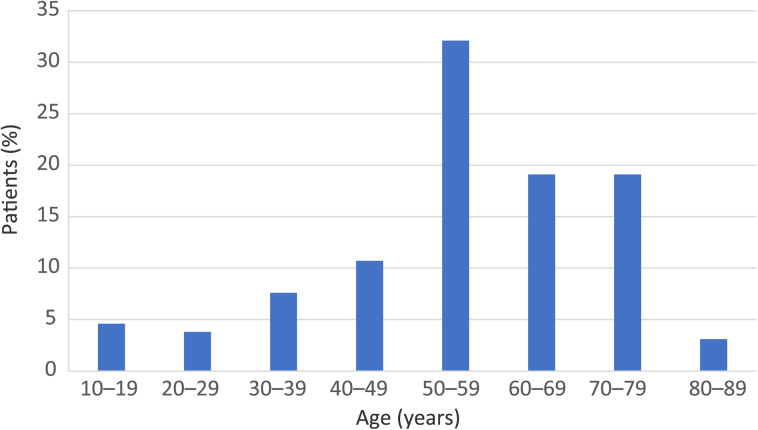
Age distribution of study cohort.

The mean age of the cohort was 55.3 years, with the youngest patient being 10 years old and the oldest being 82 years old (Figure 1).

Forty-one patients (31.3 per cent) had a radiologically normal MRI scan of the head, and 84 (64.1 per cent) had scans revealing incidental findings. These included non-specific, age-related findings such as small vessel disease (*n* = 43, 32.8 per cent), old infarcts (*n* = 13, 9.9 per cent), and cerebral or cerebellar atrophy (*n* = 17, 13.0 per cent) (Table 2). Five patients (3.8 per cent) were referred to the neurosurgical team because of incidental findings of unruptured aneurysms (*n* = 4, 3.1 per cent) and a pituitary mass (*n* = 1, 0.8 per cent). Two (1.6 per cent) of the patients were found to have changes suggestive of demyelinating disease and were referred to the neurology team (Table 2). Four patients (3.1 per cent) were found to have a benign incidental mass. These were classified as incidental because these intracranial masses, aneurysms and demyelination were located away from the olfactory pathway. Further, these findings did not exert any mass effect and were thus considered incidental findings (Table 2). One patient (0.8 per cent) was found to have a cribriform plate polyp, which was not disrupting the olfactory bulb and was hence considered an incidental finding (Table 2).

**Table 2.** MRI findings

Parameter	Cases (*n* (%))
Normal scan findings	41 (31.3)
Relevant findings	
– Significant paranasal sinus mucosal thickening*	6 (4.6)
– Olfactory neuroblastoma	0 (0)
Incidental findings	
– Insignificant paranasal sinus mucosal thickening*	50 (38.2)
– Small vessel disease	43 (32.8)
– Old intracranial infarcts	13 (9.9)
– Cerebral or cerebellar atrophy	17 (13.0)
– Aneurysms^†^	4 (3.1)
– Benign intracranial mass^‡^	5 (3.8)
– Demyelinating disease**	2 (1.6)
– Cribriform plate polyp^§^	1 (0.8)
– Paranasal sinus polyp	10 (7.6)

Cumulative frequency exceeds 100 per cent as some scans showed more than one finding. *Some patients had radiological findings of paranasal mucosal thickening. These findings were considered clinically insignificant and were not thought to contribute to their olfactory dysfunction, as previous nasoendoscopy findings were negative for mucosal disease. ^†^These were incidental unruptured aneurysms not located at areas impacting on the olfactory pathway and did not exert any mass effect on the brain. ^‡^These masses were not located at areas impacting on the olfactory pathway and did not exert any mass effect on the brain. This includes a pituitary mass (*n* = 1). **These were radiological findings suggestive of demyelinating disease. These lesions are not located at areas impacting on the olfactory pathway and did not exert any mass effect on the brain. ^§^The polyp was not disrupting the olfactory bulb. MRI = magnetic resonance imaging

Fifty patients (38.2 per cent) were found to have insignificant paranasal mucosal thickening of their sinuses (Table 2). Although these patients had radiological findings of paranasal mucosal thickening, these findings were considered clinically insignificant and were not thought to contribute to their olfactory dysfunction, as the findings of a previous nasoendoscopy were negative for mucosal disease. The insignificance is because the amount of thickening was not considered large enough to cause olfactory dysfunction, and hence, these were considered incidental findings. Ten patients (7.6 per cent) were found to have paranasal sinus polyps (Table 2). However, as with the case of mucosal disease, these findings were not considered clinically relevant because of negative nasoendoscopic findings.

Six patients (4.6 per cent) had mucosal thickening significant enough to require additional intervention (Table 2). Two (1.6 per cent) of these patients had a repeat nasoendoscopic examination, which was shown to be normal, and the patients were discharged from the clinic. One patient (0.8 per cent) was initiated on a course of oral prednisolone, and three patients (2.3 per cent) were started on a more potent nasal spray. We decided to include these patients in the ‘relevant findings’ group; however, some might consider this mucosal thickening an irrelevant finding, as the MRI scans were not performed to assess sinus mucosal disease.

No olfactory neuroblastoma was identified on the MRI scans (Table 2).

## Discussion

Of the 131 patients who underwent MRI in the five-year period, none of them demonstrated intracranial pathology that required surgical intervention. The population covered by National Health Service (NHS) Tayside was 416 550 as of 2020.^5^ In addition, NHS Tayside receives referrals from north of NHS Fife, and Fife had a population of 374 390 as of 2020.^5^ The incidence rate of olfactory neuroblastoma is 0.4 cases per million.^4^ Over the five-year period, olfactory neuroblastoma would be expected in 1.6 patients within the population. However, no olfactory neuroblastomas were identified over this period in our study. This could be because our study only examined the MRI scans of patients who presented with isolated olfactory dysfunction. These patients did not present with any other nasal or neurological signs and symptoms. It is also important to note that some of the patients may have had a computed tomography (CT) scan instead of an MRI scan because of contraindications such as cardiac pacemakers.

Most previous literature has reported low incidences of anosmia in patients with olfactory neuroblastoma, ranging from 5 per cent to 8 per cent,^6–8^ with only one study showing a 50 per cent incidence of olfactory neuroblastoma in patients presenting with anosmia or hyposmia.^6^ These studies show that olfactory dysfunction is not the main presenting complaint in those with olfactory neuroblastoma. The main complaints are nasal obstruction (50–77 per cent) and epistaxis (46–62 per cent). Other symptoms include facial pain, rhinorrhoea, and ophthalmological symptoms such as reduced visual acuity, diplopia and proptosis.^6–9^ A nasal mass was often identified on nasoendoscopic examination in patients who presented with olfactory neuroblastoma.^6,10–14^ Olfactory neuroblastomas also have a bimodal age distribution, peaking in the second and sixth decades of life.^4^ Our study population age has a mode in the fifth decade, which does not coincide with this bimodal age distribution.

Our study findings are in line with other similar studies showing a low diagnostic yield in diagnosing olfactory neuroblastoma or intracranial masses that affect the olfactory pathway.^15–17^ Across three studies, 280 MRI scans were performed, and only 2 olfactory meningiomas were identified. It is important to note that we do not know the catchment area and the population that these hospitals provide care to; hence, it is impossible to know if they are over- or under-diagnosing olfactory tumours. However, it remains that the diagnostic yield of olfactory neuroblastoma or meningiomas is low, with two of the studies, by Busaba^15^ and Hoekman *et al*.,^17^ not identifying any at all.

At present, the guidelines from the British Rhinological Society at the Royal College of Surgeons of England advise that an MRI scan should be performed if patients present with isolated anosmia for more than three months, do not have a Covid-19 infection and have normal nasoendoscopic examination findings.^18^ However, given the low diagnostic yield of identifying intracranial pathology within this study and other previous studies, the guidelines may need to be reconsidered to better utilise MRI scans in the evaluation of patients who present with isolated olfactory symptoms. These can include adopting a ‘watch and wait’ approach, to see if symptoms resolve or improve over a longer period. Further studies on the presentation of olfactory neuroblastoma may also be useful in creating risk scoring and better criteria for MRI imaging.

Our study has shown a 3.1 per cent detection rate of incidental intracranial aneurysm. This is in keeping with previous studies.^19^ These aneurysms were located in areas that did not affect the olfactory pathway, nor did they have any mass effect on the brain. These patients were referred to the neurosurgical team for further discussion. It is important to note that, on referral, these patients presented purely with olfactory dysfunction, with no other symptoms. As the purpose of the MRI scan was not to assess for incidental unruptured aneurysms, these were deemed as incidental findings. Screening, monitoring and management of incidental unruptured aneurysms is not the focus of this study and is a complicated matter, which is still being continuously studied.^20,21^ Discussion of whether performing an MRI in our patient demographic is justified because of the ability to detect incidental unruptured intracranial aneurysms will require a more in-depth study analysis and discussion.

Our study has limitations. This is a retrospective study relying on medical letters, and assessment of olfactory dysfunction is not always stated in the clinic letter or the MRI scan request form. We were unable to obtain data and perform analysis on the quantitative measurement of olfactory dysfunction, such as the University of Pennsylvania Smell Identification Test scoring system, and data were not based on an objective measure of smell. The assessment of whether or not radiological findings of mucosal thickening are significant may vary from consultant to consultant. Some may decide that one case is significant with the need for follow up or intervention, while others may decide that the same case is insignificant.

- This five-year retrospective study (*n* = 131) investigated anosmia- or dysosmia-related magnetic resonance imaging (MRI) findings, focusing on intracranial pathology
- No cases of olfactory neuroblastoma were identified
- On MRI, 41 scans (31.3 per cent) were normal, and 6 (4.6 per cent) showed significant paranasal sinus mucosal thickening
- Eighty-four (64.1 per cent) of MRI scans showed incidental findings
- The number of olfactory neuroblastoma cases identified in this study is lower than the expected detection rate of 0.4 cases per million of population

In this study, we have deemed that the majority of mucosal disease cases are clinically insignificant. A lack of endoscopic findings meant that surgical intervention would not be indicated, as the risks often outweigh the limited benefits of this surgery. Sinusitis is often self-limiting, or is managed conservatively with steroids or antibiotics if appropriate.^22,23^ Mucosal thickening and sinusitis, whether acute or chronic, can be detrimental to olfactory function. However, patients with sinusitis usually complain about nasal obstruction, nasal discharge and/or facial pain too.^24–26^ Although we have identified that mucosal disease is not the indication or the reason for performing an MRI, the MRI itself can be a form of reassurance for patients, as it helps identify the cause of the symptoms and reassures them that there is no sinister cause. Future studies could also be carried out to investigate the correlation between the severity of olfactory dysfunction and the severity and amount of paranasal mucosal thickening identified both on CT and MRI scans.

## Conclusion

Our study shows a low diagnostic yield in identifying tumours and masses that contribute to isolated olfactory dysfunction. Further studies from other health boards and on the presentation of olfactory neuroblastoma may help create better guidelines for imaging in cases of isolated olfactory dysfunction.
